# Functional evolution of the trace amine associated receptors in mammals and the loss of TAAR1 in dogs

**DOI:** 10.1186/1471-2148-10-51

**Published:** 2010-02-18

**Authors:** Eric J Vallender, Zhihua Xie, Susan V Westmoreland, Gregory M Miller

**Affiliations:** 1 New England Primate Research Center, Harvard Medical School, One Pine Hill Drive, Southborough, MA 01772, USA

## Abstract

**Background:**

The trace amine associated receptor family is a diverse array of GPCRs that arose before the first vertebrates walked on land. Trace amine associated receptor 1 (TAAR1) is a wide spectrum aminergic receptor that acts as a modulator in brain monoaminergic systems. Other trace amine associated receptors appear to relate to environmental perception and show a birth-and-death pattern in mammals similar to olfactory receptors.

**Results:**

Across mammals, avians, and amphibians, the TAAR1 gene is intact and appears to be under strong purifying selection based on rates of amino acid fixation compared to neutral mutations. We have found that in dogs it has become a pseudogene. Our analyses using a comparative genetics approach revealed that the pseudogenization event predated the emergence of the Canini tribe rather than being coincident with canine domestication. By assessing the effects of the TAAR1 agonist β-phenylethylamine on [^3^H]dopamine uptake in canine striatal synaptosomes and comparing the degree and pattern of uptake inhibition to that seen in other mammals, including TAAR1 knockout mice, wild type mice and rhesus monkey, we found that the TAAR1 pseudogenization event resulted in an uncompensated loss of function.

**Conclusion:**

The gene family has seen expansions among certain mammals, notably rodents, and reductions in others, including primates. By placing the trace amine associated receptors in an evolutionary context we can better understand their function and their potential associations with behavior and neurological disease.

## Background

Trace amine associated receptors (*TAAR*s) are a family of G-protein coupled receptors that originated prior to the emergence of jawed vertebrates [1]. The most widely studied of these receptors is TAAR1, which has been shown to bind a wide spectrum of biogenic amines and psychoactive compounds [2,3] (Additional file 1) and is a known modulator of monoaminergic activity [4]. Trace amines themselves have proven elusive to understand; the only receptors that they have been found to bind to are TAAR1 [2,3,5,6] and TAAR4 (in rat only) [2], though they also appear to be substrates at various monoamine transporters and catabolic enzymes [5-7].

TAAR1 expression in brain is observed in a variety of species including human [2], rhesus macaque [8], mouse [2], and rat [3] with its distribution widespread. Notably, expression overlaps with regions important in brain monoaminergic function and co-expression of TAAR1 and the dopamine transporter (DAT) has been observed in dopaminergic neurons [8]. Studies replacing *TAAR1 *with LacZ in knock-out mice support these findings with the staining of brain sections in these mice displaying LacZ expression throughout dopaminergic and serotonergic regions [9]. These findings, coupled with a dysregulation of trace amines in psychiatric disease [10], have made understanding the function of this gene particularly relevant.

Though the TAAR gene family is present in some form in all jawed vertebrates, the number of genes observed in any given species varies considerably [1]. While the placental mammalian ancestor is thought to have harbored nine distinct *TAAR *genes, even within this relatively recent clade there has been significant gene gain and loss; mouse, rat, and cow have added to the repertoire, while primates and dog have seen losses [1,11,12]. Functional work on members of the *TAAR *gene family other than TAAR1 is sparse, but what has been done suggests that other TAAR gene products do not bind the traditional TAAR1 ligands (e.g., β-phenylethylamine (β-PEA)) [2,13], but instead show a distinct ligand set related to environmental perception [14]. Expression studies fail to find broad expression of these other TAAR family members in the brain but rather observe localization predominantly in the olfactory apparatus [1,14].

This functional dichotomy between TAAR1 and its cousins is significant. While other members of the *TAAR *gene family have seen recurrent pseudogenization and duplication, *TAAR1 *has been evolutionarily stable. *TAAR1 *was the first to arise and remains the only *TAAR *gene present in every species studied with the possible exception of the neotelost fish who nevertheless harbors another, evolutionarily similar, *TAAR1 *cousin [1,15]. Yet, despite this conservation, *TAAR1 *shows sequence divergence across species and species-specific pharmacological profiles with drug potency (EC_50_) differences of 10-fold or more common [1,11,16,17]. While it remains unclear what practical effect these differences have *in vivo*, it is noteworthy that this variation exists, despite the conservation of the gene itself and its unique evolutionary history.

In order to better understand the evolutionary context in which this receptor family evolved and to better grasp its likely functional significance we have explored the relationship of various *TAAR *homologs in twenty mammals, a marsupial, a monotreme and an outgroup avian. We have characterized the gain and loss of *TAAR *homologs among mammals and performed evolutionary analyses to better understand the selective constraints under which they operate. We have also more fully investigated the evolutionary history of *TAAR1 *with a focus on the carnivores and the functional implications of a loss of *TAAR1 *in dogs.

## Results and Discussion

Initially we gathered all reported *TAAR *homologs from eleven species for whom annotated genomic sequence was available (*Mus musculus*, mouse; *Rattus norvegicus*, rat; *Pan troglodytes*; chimpanzee; *Homo sapiens*, human; *Bos taurus*, cow; *Sus scrofa*, pig; *Equus caballus*, horse;*Canis familiaris*, dog; *Monodelphis domesticus*, opossum; *Ornithorhynchus anatinus*, platypus; and *Gallus gallus*, chicken. We also amplified and sequenced *TAAR *homologs from three new world monkey species (*Callithrix jacchus*, common marmoset; *Saguinus oedipus*, cotton-top tamarin; and *Saimiri sciureus*, squirrel monkey), sequence confirmed the genomic data available for rhesus macaque, *Macaca mulatta*, and identified putative orangutan (*Pongo pygmaeus*) homologs through trans-alignments with the as yet unannotated genomic sequence.

**Figure 1:**
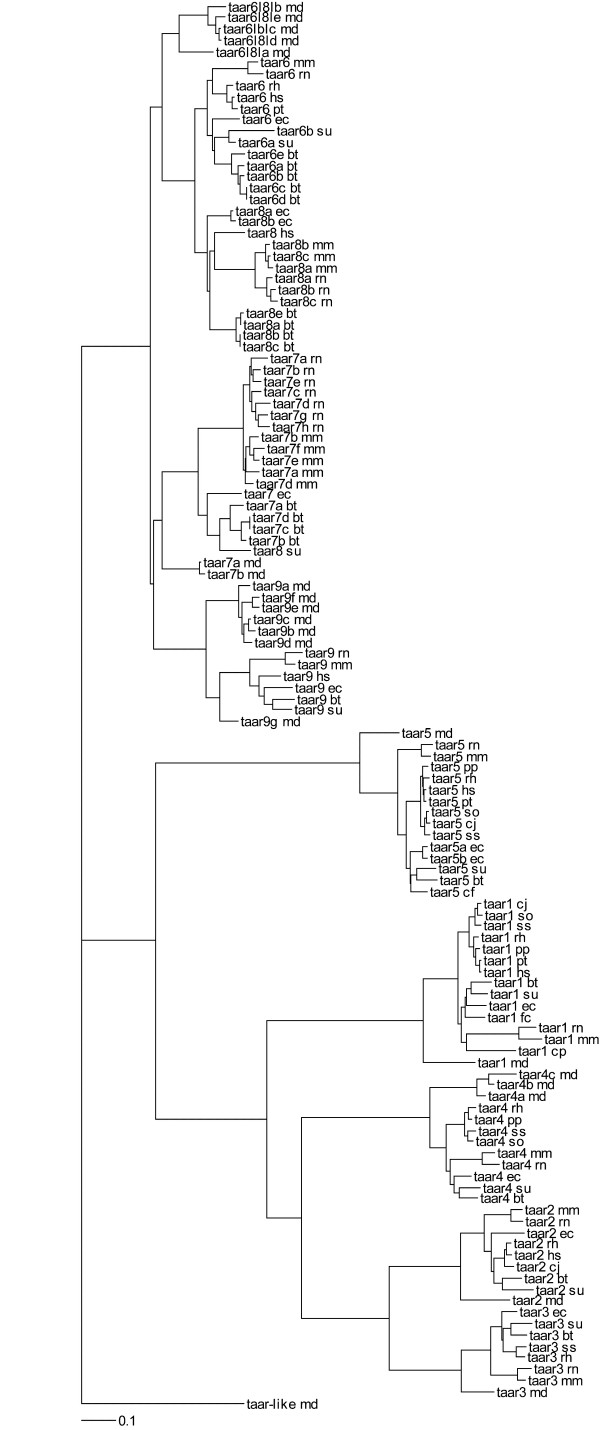
**Phylogeny of mammalian TAAR genes**. Branch length is proportional to nucleotide divergence. d_N_/d_S _ratios for all branches can be found in Additional file 1.

Computationally annotated genomic sequences are currently focused on identifying intact genes and exclude pseudogenes. As such, for many of the species for whom genomic sequences were relied upon only whole and intact genes were identified. (Though it is noted that even this set is incomplete do to errors and incomplete data in the annotation process [18].) We then aligned, in frame, the identified genes, calculated evolutionary parameters and generated a phylogenetic tree using RAxML [19]. This resulting phylogram largely mirrors the previously identified relationships between the trace amine associated receptors [1,11] and species relationships (Figure 1, Additional File 2). The incongruencies that exist are simply explained by a paucity of mutations reflective of a small physical region and short evolutionary time. Beyond the nine ancestral *TAAR*s, expansions have been species specific (symparalogs) and we observe no evidence for paralogs shared between species (alloparalogs). However, *TAAR6 *and *TAAR8 *in placental mammals themselves appear to be alloparalogous sharing a single ancestral ortholog with marsupials. This ancestor of mammalian *TAAR6 *and *TAAR8 *we tentatively call *TAAR6L8L *(trace amine associated receptor 6-like, 8-like) and has seen apparent duplications in the opossum lineage. We further identify an opossum specific gene that appears to serve as an outgroup to all *TAAR*s, tentatively called *TAARL *(trace amine associated receptor-like). Further investigation of these marsupial *TAAR *genes will be required to understand their functional and evolutionary relevance.

We also considered the relative rate of amino acid fixation (d_N_/d_S_) among the lineages. Without exception, all lineages showed d_N_/d_S _values less than one indicative of negative, purifying, selection (see Additional file 3). Because of the observed differences between ligands and expression patterns associated with TAAR1 as compared to other family members, we performed a branches test to identify if there was a significant difference between the d_N_/d_S _values among TAAR1 genes compared to other family members. Interestingly there was a significant difference (p < 0.001) but with TAAR1 showing an elevated, though still purifying, d_N_/d_S _of 0.256 compared to the remainder of the family at a d_N_/d_S _of 0.164. We compared this to the TAAR5 clade which also is robustly conserved without pseudogenization. TAAR5 is evolutionarily statistically indistinguishable from other TAAR family members.

**Figure 2:**
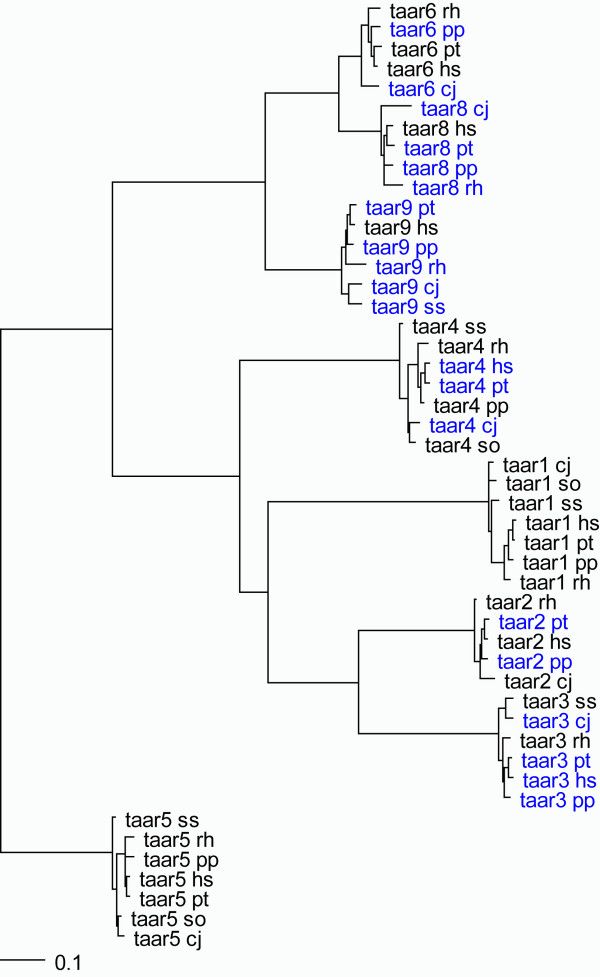
**Phylogeny of primate TAAR genes**. Branch length is proportional to nucleotide divergence. Pseudogenes are identified in blue.

To more thoroughly investigate the gain and loss of *TAAR *genes and their relevance to human behavior and disease, we interrogated the entire family repertoire of humans, chimpanzees, orangutans, rhesus macaques, and marmosets as well as tamarin and squirrel monkey sequences for which amplicons were successful. Previous studies had noted numerous pseudogenization events in the evolution of human and chimpanzee *TAAR*s [10] and this finding appeared to be generalized across primates with pseudogenes common among orangutans, rhesus macaques, and marmosets as well. A tree depicting the relationships among primate *TAAR*s is shown in Figure 2 with pseudogenes noted. The data remains too incomplete to adequately model birth-and-death processes, however, and will likely require more complete identification of entire *TAAR *gene complements among all mammals for conclusion. Two genes, however, are conserved and intact among all five primate species, *TAAR1 *and *TAAR5*. These findings suggest these genes explicitly as the most likely to be implicated in disease phenotypes among the gene family.

**Figure 3:**
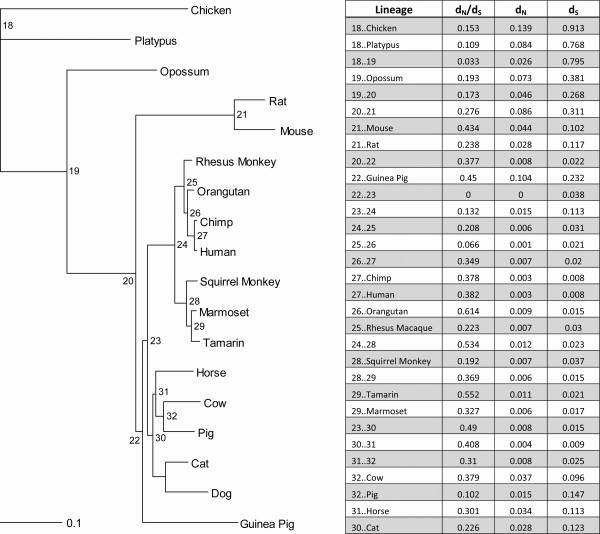
**Phylogeny of mammalian TAAR1**. Branch length is proportional to nucleotide divergence. Lineage-specific d_N_/d_S _values are displayed. Note that the d_N_/d_S _value associated with the cat terminal branch extends to the last common ancestor between *Carnivora *and *Cetartiodactyla*.

*TAAR1 *and *TAAR5 *also seem to be the most conserved broadly across all the species studied, but while *TAAR5 *is intact for all mammalian species in which it has been identified, our interrogations of the published dog genomes found *TAAR1 *to be a pseudogene. Indeed, complete and conserved coding regions for *TAAR1 *were identified for all mammals under study with the exception of the dog which had several frame-shift mutations apparently rendering it a pseudogene. In-frame insertion/deletions can be seen in several species (mouse, rat, chicken, and cat), but these are all localized to the third intracellular domain. Overall divergence values for the gene across all species are broadly representative of expected mutation rates. Using maximum likelihood based methods we reconstructed putative ancestral sequences and probed the tree for evidence of positive selection. As shown in Figure 3, d_N_/d_S _values for all branches were below 1 indicative of negative selection at work on the gene. Lineages that displayed even moderately higher d_N_/d_S _values tended to be shorter and indicative more of stochastic noise rather than obscured signal. Further, there was no evidence of positive selection at either the level of individual codons. The third intracellular domain and the third extracellular domain showed moderately higher levels of amino acid divergence as compared with other regions in the gene, though still without approaching levels anticipated through positive selection (data not shown). This is perhaps unsurprising given that among TAAR family genes as a whole these regions tend to be the least conserved [1].

**Figure 4:**
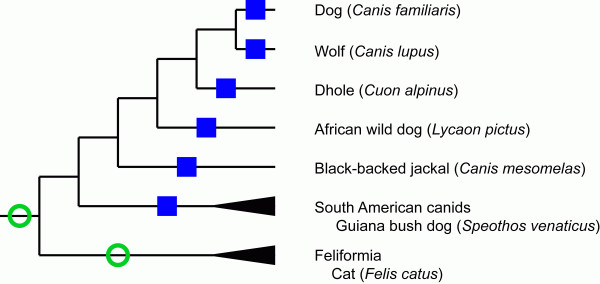
**Simplified phylogeny of *Carnivora***. Green (open) circles denote *TAAR1 *open reading frames. Blue (closed) squares denote *TAAR1 *pseudogenes.

While TAAR1 is under purifying selection across a diverse group of mammals, this does not appear to be the case for dog. Sequence obtained from the dog genome shows several frame shift mutations in the orthologous *TAAR1 *region. Dog genomes produced by public and private efforts on two different breeds of dog, boxer and poodle showed no differences between the breeds, nor did we find any difference when we separately amplified and sequenced the dog *TAAR1 *from genomic DNA isolated from MDCK cells, a cell line derived from the kidney of a cocker spaniel. Further interrogation of the dog genome using feline, murine, or human *TAAR1 *confirmed the orthology of the canine *TAAR1 *pseudogene and failed to find evidence of a duplication event. To determine whether the loss of *TAAR1 *was coincident with the canine domestication event, we also sequenced *TAAR1 *from three wild gray wolf populations, but again found identical sequences to those from domesticated dog. Guided by carnivore phylogeny [20], we surveyed *TAAR1 *in four additional caniform carnivores. Wolf-like canids (Chinese dhole, African wild dog, and black-backed jackal) and South American canids (bush dog) showed the same pseudogenization markers as was observed for the dog and wolf. Because *TAAR1 *is intact in cats, the pseudogenization event seems to have occurred prior to the divergence of the tribe Canini but subsequent to the divergence from the feliforms (Figure 4).

While *TAAR1 *is inactivated in dogs, it is not certain that its function has been lost. While analysis of the dog genome failed to identify a specific recent duplication event of *TAAR1*, it did identify two other TAAR family members with intact coding regions and so it is conceivable, if unlikely, that a compensatory functional change may have appeared. It is also notable that the dog genome, in its current state, remains incomplete. While the *TAAR1 *genomic region maps to a chromosome with flanking genes (*VNN1 *upstream and *STX7 *downstream) identical to the genes that flank the TAAR regions in other species, *TAAR2*, *TAAR4*, and *TAAR5 *map to a contig that has not been assembled onto a chromosome. *TAAR3*, *TAAR6*, *TAAR7*, *TAAR8*, and *TAAR9 *homologs are not readily apparent in the dog genome though several large gaps exist in the region downstream of the *TAAR2*/*4*/*5 *cluster where *TAAR6*/*7*/*8*/*9 *would be expected. In all the dog genome assemblies, the *TAAR *locus is far from finished quality. Because of this we sought to determine whether TAAR1 functionality, if not TAAR1 itself, could be observed in dogs.

**Figure 5:**
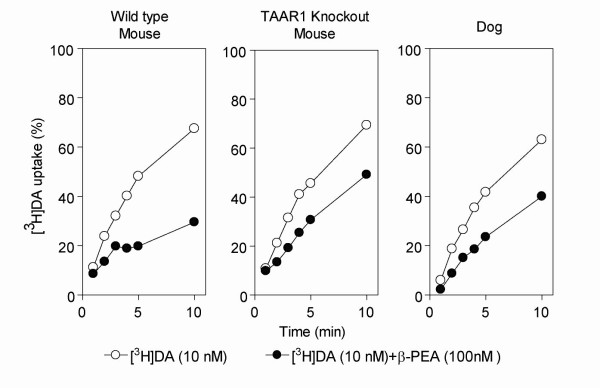
**Modulation of [^3^H]dopamine uptake by β-PEA in brain striatal synaptosomes**. Synaptosomes from wild-type mouse (n = 3), TAAR1 knockout mouse (n = 3) and dog (n = 1) were exposed to 10 nM [^3^H]dopamine alone or 10 nM [^3^H]dopamine plus 100 nM β-PEA for the indicated times. Uptake values are percentage of the maximal uptake. Note the reduction in dopamine uptake during β-PEA challenge in wild-type mouse as compared to TAAR1 knockout mouse and dog. Data are presented as mean ± S.E.M.

To do so, we used an established assay for TAAR1 functionality in brain synaptosomes that distinguishes between the presence and absence of a functional TAAR1 receptor [4,8,21]. The assay uses 10 nM [^3^H]dopamine as a tracer combined with 100 nM β-PEA as the TAAR1 agonist [21]. An enhanced uptake inhibition by DAT occurs in TAAR1 and DAT co-transfected cell lines or striatal synaptosomes where TAAR1 is present, but not in cell lines without TAAR1 or in synaptosomes derived from TAAR1 knock-out mice. Notably, this response is robust to species differences, with similar results shown in cell lines, old world and new world monkey synaptosomes, and mouse synaptosomes [4]. Here, we show that in fresh striatal brain synaptosomes from dog, wild-type mouse and *TAAR1 *knock-out mouse, 10 nM [^3^H]dopamine alone or 10 nM [^3^H]dopamine and 100 nM β-PEA was taken up specifically by DAT over time. Consistent with our previous findings [4,8,21], synaptosomes prepared from the wild-type mouse showed an enhanced [^3^H]dopamine uptake inhibition initiating after 3 minutes when β-PEA was present, while this effect was not observed in synaptosomes prepared from TAAR1 knock-out mice. Synaptosomes derived from dog striatum responded to the treatment similar to those prepared from the TAAR1 knock-out mice, strongly suggesting the functional absence of TAAR1 (Figure 5).

Two groups have separately created *TAAR1 *knockout mice and not only are these mice fully viable, but the mice show no gross physical or behavioral abnormalities and identification of any non-pharmacologically induced phenotype has not been forthcoming [9,22]. Yet the conservation of the gene for the last 450 million years across numerous species belies an importance. Concurrent with a role as a monoaminergic modulator, knock-out mice show changes in their dopaminergic system compared to wild types including increased high-affinity dopamine receptor D_2_[22] and higher neuronal firing rates in the ventral tegmental area [9,22], which is important in brain reward circuitry [9]. It may be noteworthy then that while the D_2 _receptor antagonist pimozide does not change the self-administration of β-PEA in dogs [23], it does block β-PEA induced locomotion in mice [24]. Further, while the self-administration of β-PEA in dogs is unaffected by the D_2 _antagonist, it increases self-administration of amphetamine both in dogs [25] and rats [26].

## Conclusion

Our understanding of neuroaminergic signaling has recently become more complicated with the emergence of the TAAR gene family. These genes represent a heretofore unexplored mechanism of neuromodulation. They have gained a further importance with findings suggesting an association with schizophrenia [27,28]. In these studies we catalog the TAAR family members across mammals, demonstrating a pattern of pseudogenization and duplication among most of the family members. In primates particularly, only two members, TAAR1 and TAAR5, are completely conserved, suggesting perhaps a greater importance for these genes. Yet despite this evolutionary flux there seems to be a strong purifying selection across all intact genes.

Both because of its conservation as well as the fact that only it binds neurotransmitters, TAAR1 has proven particularly interesting. Indeed TAAR1 knock-out mouse may show some of the same endophenotypes as schizophrenic patients [22] and have been touted as a model system. Yet here we demonstrate that there exist naturally occurring TAAR1 knockouts: dogs and their close relatives. Among these species there does not appear to be an intact open reading frame and *ex vivo *studies show effects similar to those seen in *TAAR1 *knockout mice. Yet it remains unclear what, if any effects, this loss of function has had. While it is tantalizing to speculate that many behavioral characteristics of dogs, including their relative ease of training, may involve reward encoding and perception, and suggest that this is related to TAAR1 effects on the dopamine system, it remains unproven. Indeed, any association between dog behavior and TAAR1 functional loss must also account for the other Canini species.

That dogs and their brethren can well tolerate the loss of TAAR1 despite its evolutionary conservation must provoke questions as to the relevance of the gene to schizophrenia or other mental health diseases. While it has been suggested that perhaps another member of the trace amine associated receptor family is contributory to the schizophrenic phenotype [28], the widespread loss of *TAAR *genes suggests an evolutionary lability that seems contraindicative. Rather the ligand binding studies suggest a dichotomy between TAAR1 and the remainder of the family that is broadly supported by the observed differences in gene phylogenies. The suggestion from rodents that TAAR family members other than TAAR1 are related to chemosensory perception [14] is bolstered by these findings as well. The patterns of species-specific duplications and pseudogenization events are reminiscent of that seen for olfactory genes [29].

This work demonstrates the strengths of applying evolutionary studies to functional analyses. From the phylogenies of the TAAR genes, TAAR1 appears qualitatively different in its pattern of gene gain and loss. This coupled with our knowledge of TAAR ligand binding suggests a dichotomy of function among the receptors. At the same time the tolerance of loss of TAAR1 in dogs offers a warning for association studies with disease; the subtleties of effect must be considered. Understanding the paradox of evolutionarily ancient conservation and an accommodation of recent loss will shed significant light on neuroaminergic biology and the genetic basis underlying brain and behavior.

## Methods

**Figure 6:**
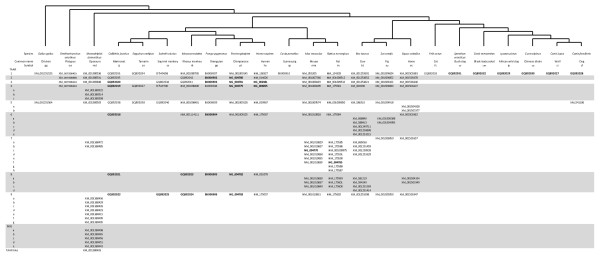
**Table depicting the Genbank accession numbers for all the genes used in this study**. A cladogram depicting species relationships is presented above the table. Pseudogenes are in bold.

TAAR1 sequences from *Mus musculus*, mouse; *Rattus norvegicus*, rat; *Cavia porcellus*, guinea pig; *Pan troglodytes*; chimpanzee; *Homo sapiens*, human; *Pongo pygmaeus*, orangutan; *Felis catus*, cat; *Bos taurus*, cow; *Sus scrofa*, pig; *Equus caballus*, horse;*Monodelphis domesticus*, opossum; *Ornithorhynchus anatinus*, platypus; and *Gallus gallus*, chicken, were obtained from genomic databases. Likewise, dog, *Canis familiaris*, TAAR1 sequences for both the boxer (public genome) and poodle (Celera genome) were obtained bioinformatically. Genomic DNA was obtained from the cocker spaniel-derived MDCK cell line, and blood drawn from *Macaca mulatta*, rhesus macaque; *Callithrix jacchus*, common marmoset; *Saguinus oedipus*, cotton-top tamarin; and *Saimiri sciureus*, squirrel monkey. Genomic DNA was isolated using the Flexigene kit (Qiagen, Valencia, CA) following manufacturer's protocols. DNA from three wild wolf (*Canis lupus*) populations, Yellowstone National Park in the United States, Canadian Rockies, and Spain, were kindly provided by RK Wayne. Black-backed jackal, *Canis mesomelas*; Chinese dhole, *Cuon alpinus*; African hunting dog, *Lycaon pictus*; and bush dog, *Speothos venaticus *samples were obtained with the help of OA Ryder and the San Diego Zoo. Genbank accession numbers for all genes presented in this manuscript and a species cladogram for all species used in the present study are available in Figure 6.

TAAR1 was amplified from genomic DNA by trial-and-error using primers designed against the dog genome as well as conserved regions between the dog and cat genomes. Following verification of product size and specificity on a 1.5% agarose gel, the PCR products were purified using the QIAquick Gel Extraction Kit (Qiagen) according to manufacturer's protocol. Sequencing was performed using the CEQ8000 Genetic Analysis System and CEQ DTSC Quick Start Kit (Beckman Coulter). For all species under study, both strands were sequenced and analyzed using Vector NTI (Invitrogen).

*TAAR1 *knockout mice and wild type mice were derived from six pairs of heterozygous mice given to us as a gift by Lundbeck Research USA, Inc[22]. All procedures were conducted in accordance with the Animal Experimentation Protocol approved by the Harvard Medical Area Standing Committee on Animals. Fresh post-mortem brain tissue from mongrel dog sacrificed for unrelated purposes was obtained with the help of M. Contreras, N. McDannold, N. Vykhodtseva and Y. Zhang at the Harvard Medical School. *TAAR1 *knockout and wild-type mouse brain tissue was collected from our mouse colony at the New England Primate Research Center (Southborough, MA). Dog striatum offered enough tissue to allow preparation from a single animal while mouse tissues were pooled. All procedures were conducted in accordance with the Animal Experimentation Protocol approved by the Harvard Medical Area Standing Committee on Animals. Fresh tissues were homogenized in 1.5-ml Eppendorf centrifuge tubes with 10× volume of ice-cold unbuffered 0.32 M sucrose solution (pH 7.0), using a motor-driven pellet pestle. The homogenate was centrifuged (1000 *g*, 10 min at 4°C) to yield a crude nuclear pellet and low-speed supernatant. The low-speed supernatant fraction was carefully transferred into another fresh tube and centrifuged at 10,000 *g *and 4°C for 20 min to yield a synaptosome-containing pellet. The resulting pellet was resuspended in an appropriate volume of ice-cold uptake buffer (a modified Krebs buffer: 25 mM HEPES, 120 mM NaCl, 5 mM KCl, 2.5 mM CaCl_2_, 1.2 mM MgSO_4_, 1 μM pargyline, 2 mg/ml glucose, and 0.2 mg/ml ascorbic acid, pH 7.5) for further assays.

50 uL of the synaptosome preparation was added into 1.5-ml Eppendorf centrifuge tubes and exposed to [^3^H]dopamine (10 nM; 60 Ci/mmol; PerkinElmer Life and Analytical Sciences) only or combined with 100 nM β-PEA at 25°C in uptake buffer for various times as indicated. The uptake by the synaptosomes at 30 min in [^3^H]dopamine only was taken as maximal uptake (100%). Non-specific uptake was defined in the presence of 10 μM methylphenidate. Uptake reactions were terminated by addition of 1 ml of ice-cold uptake buffer into the tubes and immediate centrifugation at 1,000 g (for cells) or 10,000 g (for synaptosomes) at 4°C for 3 min. The resulting pellets were rinsed twice with 1 ml of ice-cold uptake buffer and then incubated in 1× PLB buffer for 30 min on a shaking platform at 200 rpm, prior to being transferred into scintillation vials containing 4 ml of Beckman ReadySafe scintillation cocktail and counted on a Beckman LS6000IC scintillation spectrophotometer for 1 min/sample.

For studies of intact genes, nucleotide sequences were aligned in-frame using ClustalW, while alignments including pseudogenes were made directly from nucleotide sequences[30]. Trees were generated with RAxML [19,31,32] using a maximum likelihood inference and validation with 1000 bootstrap analyses. CAFE[33] was used to attempt to model the birth and death processes in primates with a maximum likelihood value of λ, probability of gene birth or death per million years, calculated as 0.0141. However with existing data the distribution of pseudogenes was not significantly different than expected. d_N_/d_S _(K_A_/K_S_) values for protein-coding sequences were calculated using PAML (using model 1, free ratio, in codeml) as well as the Li et al. [34] and Yang and Neilsen [35] methods with no significant differences observed.

PAML was used to test for branch-specific evolution in codeml. The parameters are shown below in Additional file 4. Specifically we tested the hypotheses that either the TAAR1 clade or TAAR5 clade of Figure 1 had a significantly different d_N_/d_S _ratio than the remainder of the tree. Significance values were obtained by comparing the likelihood ratio statistics (2 Δℓ) to a  with degrees of freedom equal to the difference between number of parameters in the model. PAML was also used to test site-specific evolution. Parameters for site-specific evolution are shown in Additional file 5. The hypothesis tested whether there were any sites under positive selection in TAAR1. Significance was calculated as above.

## Authors' contributions

EJV and GMM designed the study. SVW performed necropsies and collected tissue. ZX generated synaptosomes and preformed uptake assays. EJV preformed analyses of genetic data. All authors contributed to the final manuscript.

## Supplementary Material

Additional file 1 **Table S1.** Representative ligands for TAAR1. Chemical structures are shown associated with letter following compound. Species for which binding of specific ligands has been demonstrated are shown (reviewed in Zucchi et al., 2006 [6]). Click here for file

Additional file 2 **Enlarged phylogeny of mammalian TAAR genes with labeled internal nodes**. Additional File 1 is an enlarged version of Figure 1 with internal nodes labeled for ease of interpretation. Click here for file

Additional file 3 **Table S2.** d_N_, d_S_, and d_N_/d_S _values associated with Figure 1. Internal node assignment can be more easily viewed in Supplementary Figure 1. Click here for file

Additional file 4**Table S3.** Log likelihood values and parameter estimates in branch models test. p, number of ω parameters in model. ω_0_, ω_TAAR1_, ω_TAAR5_, d_N_/d_S _values from background, TAAR1 clade, and TAAR5 clade respectively.  estimated transition-transversion ratio. ℓ, log-likelihood valueClick here for file

Additional file 5**Table S4.** Log likelihood values and parameter estimates in sites models test. p, number of parameters in model.  estimated transition-transversion ratio. ℓ, log-likelihood value. , estimated proportion of class *n *sites. , estimated d_N_/d_S _of class *n *sites. *p *and *q*, beta fit parameters.Click here for file
