# Supplementary figures and images for: Functional evolution of the trace amine associated receptors in mammals and the loss of TAAR1 in dogs
Source: BMC Evol Biol. 2010 Feb 18;10:51. doi: 10.1186/1471-2148-10-51 (PMC2838891; doi:10.1186/1471-2148-10-51)

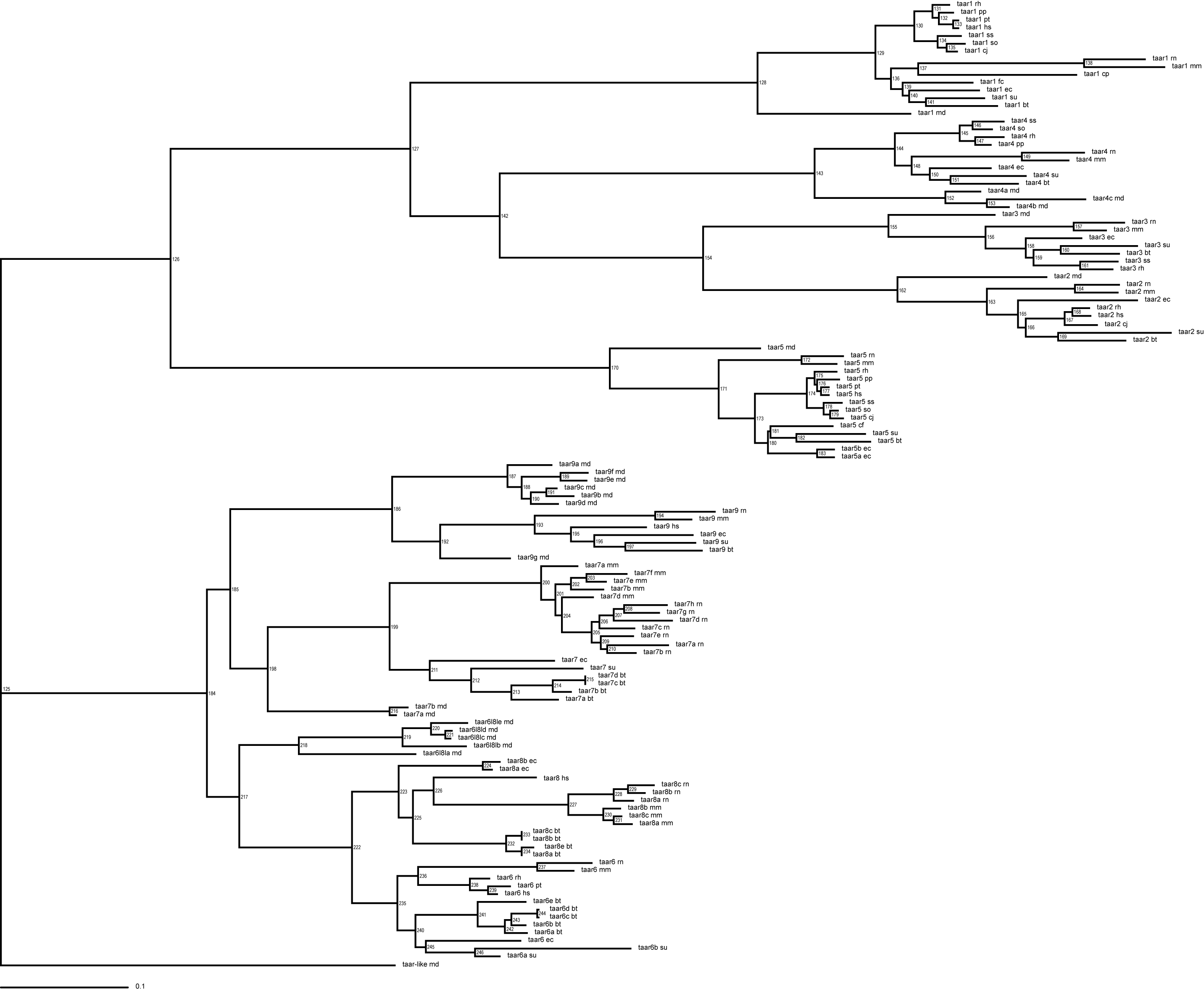

Supplement: Additional file 2 — Enlarged phylogeny of mammalian TAAR genes with labeled internal nodes. Additional File 1 is an enlarged version of Figure 1 with internal nodes labeled for ease of interpretation. [file 1471-2148-10-51-S2.TIFF]
